# Anti-BCMA CAR T administration in a relapsed and refractory multiple myeloma patient after COVID-19 infection: a case report

**DOI:** 10.1186/s13256-020-02598-0

**Published:** 2021-02-19

**Authors:** D. Madduri, S. Parekh, T. B. Campbell, F. Neumann, F. Petrocca, S. Jagannath

**Affiliations:** 1 Department of Hematology and Medical Oncology, Tisch Cancer Institute, Icahn School of Medicine at Mount Sinai, 1 Gustave L Levy Pl, Box 1185, New York, NY 10029 USA; 2 Bristol-Myers Squibb, Princeton, NJ USA; 3 bluebird bio, Cambridge, MA USA

**Keywords:** COVID-19, SARS-CoV-2, CAR T cell, Multiple myeloma, Case report

## Abstract

**Background:**

Very little is known about the risk that severe acute respiratory syndrome coronavirus 2 (SARS-CoV-2) viral infection poses to cancer patients, many of whom are immune compromised causing them to be more susceptible to a host of infections. As a precautionary measure, many clinical studies halted enrollment during the initial surge of the global Novel Coronavirus Disease (COVID-19) pandemic. In this case report, we detail the successful treatment of a relapsed and refractory multiple myeloma (MM) patient treated with an anti-B cell maturation antigen (BCMA) chimeric antigen receptor (CAR) T cell therapy immediately following clinical recovery from COVID-19.

**Case presentation:**

The 57 year old Caucasian male patient had a 4-year history of MM and was considered penta-refractory upon presentation for CAR T cell therapy. He had a history of immunosuppression and received one dose of lymphodepleting chemotherapy (LDC) the day prior to COVID-19 diagnosis; this patient was able to mount a substantial immune response against the SARS-CoV-2 virus, and antiviral antibodies remain detectable 2 months after receiving anti-BCMA CAR T cell therapy. The recent SARS-CoV-2 infection in this patient did not exacerbate CAR T-associated cytokine release syndrome (CRS) and conversely the CAR T cell therapy did not result in COVID-19-related complications. One month after CAR T cell infusion, the patient was assessed to have an unconfirmed partial response per International Myeloma Working Group (IMWG) criteria.

**Conclusion:**

Our case adds important context around treatment choice for MM patients in the era of COVID-19 and whether CAR T therapy can be administered to patients who have recovered from COVID-19. As the COVID-19 global pandemic continues, the decision of whether to proceed with CAR T cell therapy will require extensive discussion weighing the potential risks and benefits of therapy. This case suggests that it is possible to successfully complete anti-BCMA CAR T cell therapy after recovery from COVID-19.

CRB-402 study registered 6 September 2017 at clinicaltrials.gov (NCT03274219).

## Background

The global COVID-19 pandemic represents a worldwide public health crisis and directly impacts cancer care. Patients with multiple myeloma (MM) have cellular and humoral immune dysfunction causing them to be more susceptible to infections [1, 2]. Anti-B cell maturation antigen (BCMA) chimeric antigen receptor (CAR) T cell therapy is emerging as a promising option for relapsed myeloma patients; however, most clinical trials of CAR T therapy for MM were paused during the pandemic because of the possibility of increased morbidity and mortality with COVID-19. Specifically, in MM patients, it is unclear whether the immunosuppression resulting from conditioning regimens used with CAR T cell therapy may pose an increased risk of infection with severe acute respiratory syndrome coronavirus 2 (SARS-CoV-2). In addition, COVID-19 may trigger an inflammatory cascade [3-5] similar to the cytokine release syndrome (CRS) seen in some patients treated with CAR T cells [6]. Our experience in MM patients with COVID-19 showed they have a similar mortality compared to the general age-matched COVID-19-infected population [7]. Our practice has therefore been to weigh the risks and benefits of treatment to tailor therapy for individual MM patients during the COVID-19 pandemic. Here, we report the first case to our knowledge of an MM patient safely treated with anti-BCMA CAR T cell therapy immediately after clinical recovery from COVID-19.

## Case presentation

A 57-year-old Caucasian male patient with a 4-year history of IgG-kappa MM was referred to Mount Sinai Hospital in New York City in early February 2020 because of disease progression. He was penta-refractory (refractory to two proteasome inhibitors, two immunomodulatory agents, and an anti-CD38 antibody) and had previously received nine lines of therapy.

In early February 2020, approximately 3.5 weeks prior to the first confirmed case of COVID-19 in New York City, the patient was enrolled in a clinical study (NCT03274219) of bb21217, an investigational BCMA-directed CAR T cell therapy. The study was conducted in accordance with the Declaration of Helsinki and International Conference on Harmonisation guidelines for Good Clinical Practice, and the protocol was approved by local or independent institutional review boards (IRB) at each study center. Informed consent was obtained from each patient. The patient received bridging therapy with melphalan and bortezomib while awaiting CAR T cell manufacturing. He was asymptomatic and screened negative by PCR test for SARS-CoV-2 two days prior to a planned 3-day course of lymphodepleting chemotherapy (LDC). Approximately 24 hours after receiving the first dose of LDC [cyclophosphamide (300 mg/m^2^)/fludarabine (30 mg/m^2^)], the patient returned to clinic with fever, cough, and diarrhea. Nasopharyngeal polymerase chain reaction (PCR) test confirmed SARS-CoV-2 infection.

CAR T infusion was held in the setting of active COVID-19 infection, and the patient was admitted to the hospital for observation. He was afebrile upon admission with a mild cough that resolved within 1 day. He was given granulocyte colony stimulating factor (G-CSF) for grade 1 neutropenia and discharged with instructions to self-isolate at home after 3 days of hospitalization.

**Fig. 1:**
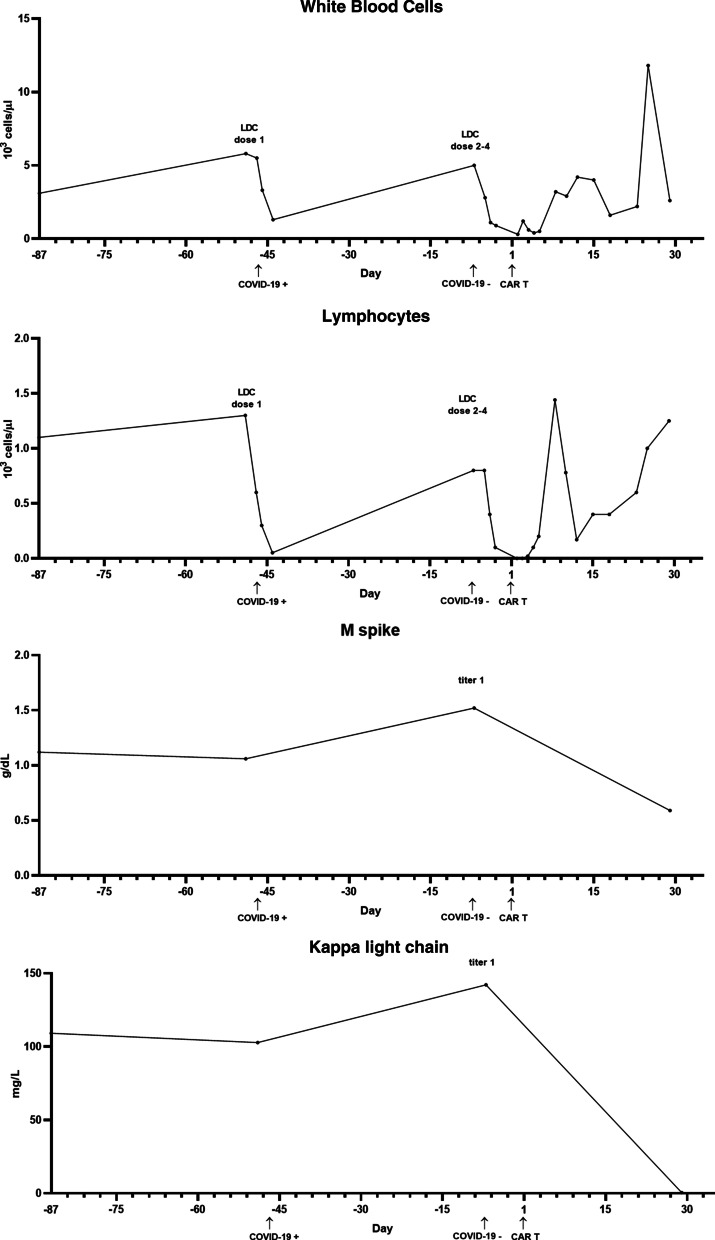
Clinical course: blood laboratory values from initial baseline assessment through 30 days post CAR T cell administration. LDC was administered on days -47 and days -5 through -3; positive COVID-19 test by PCR day -46, negative COVID-19 test by PCR day -7; CAR T cell administration day 1, anti-viral antibody titer day -7. Repeat anti-viral antibody titers were conducted day 43 and day 73 (not shown)

**Fig. 2:**
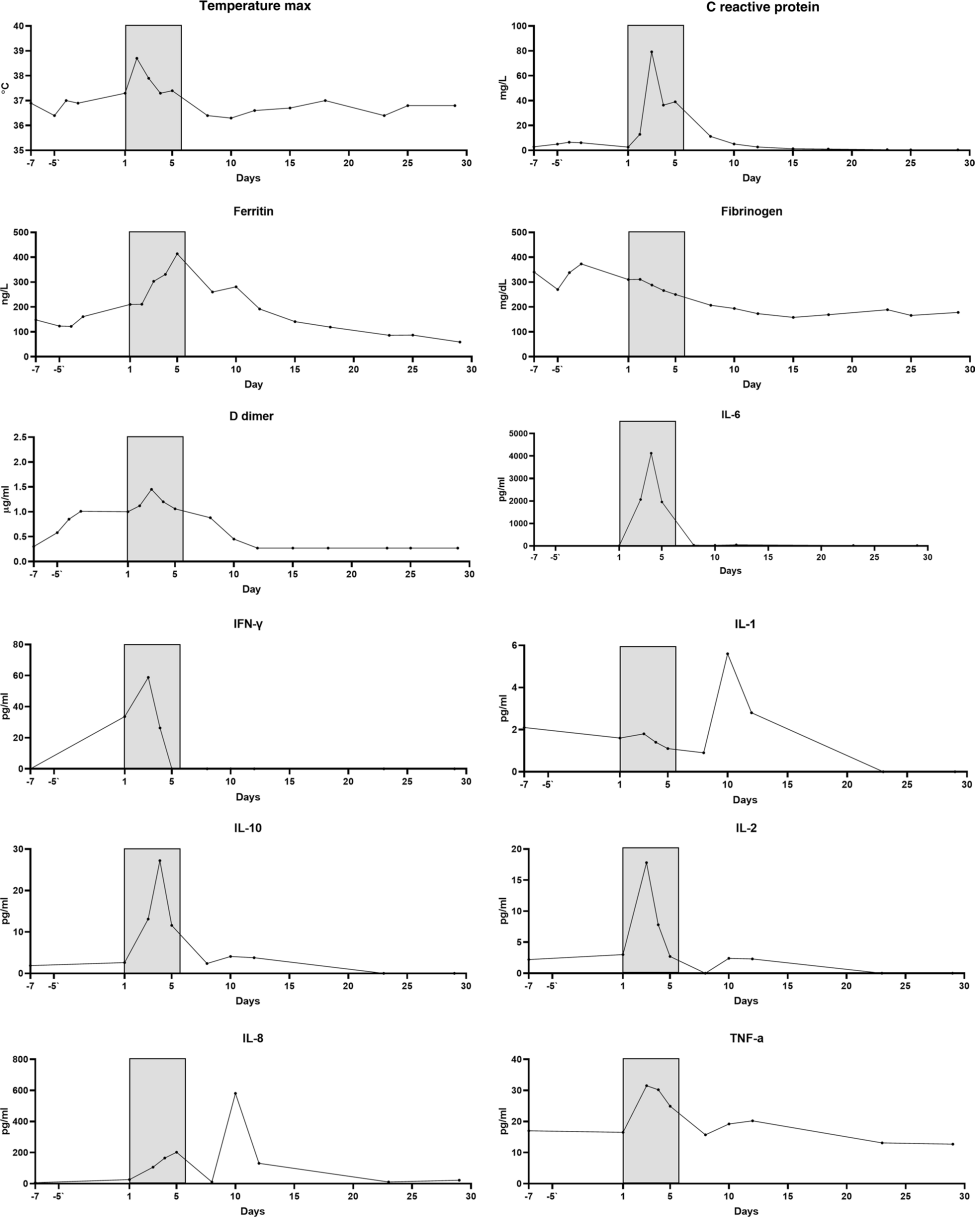
Cytokine release syndrome course and recovery: temperature, inflammatory and cytokine values from CAR T cell administration through 30 days post CAR T cell administration, CRS course (shaded) days 1–6

The patient was monitored weekly, and further therapy was held until SARS-CoV-2 clearance was confirmed by nasopharyngeal PCR test, 39 days after COVID-19 diagnosis. Upon approval by the sponsor and our IRB, the patient reinitiated the full 3-day course of LDC in preparation for CAR T cell administration. At this point, inflammatory markers were normal, and SARS-CoV-2 antibodies were detected at a titer of 1:2880 using an IgG assay developed by Mount Sinai [8]. On the day of CAR T cell infusion, lymphocytes in the peripheral blood were undetectable and the patient showed profound leukopenia (Figure 1). Twelve hours after CAR T cell infusion, clinical signs consistent with Grade 1 CRS developed, including fever and tachycardia [9]. On day 2, CRS escalated to Grade 2 and was accompanied by hypotension (81/52), only transiently responsive to fluids; thus, tocilizumab (8 mg/kg) × 1 was given. The patient experienced 2 more days of low-grade fever (Figure 2) and grade 1 hypotension. CRS resolved by day 6, and cytokines returned to pretreatment levels by day 9 (Figure 2). Blood counts improved by day 12 except lymphopenia, which persisted through day 14 (Figure 1). Based on 1-month follow-up post-CAR T cell infusion, the patient did not experience other complications, remained SARS-CoV-2 negative, and showed normalization of free kappa light chain with a 61% decrease in serum M protein (Figure 1), consistent with partial response per international myeloma working group (IMWG) criteria. Repeat SARS-CoV-2 antibody titer at his 1-month follow up was 1:960 and at 2-month follow up was 1:320.

## Discussion/Conclusions

Our case adds important context to the evolving literature about treatment of MM patients in the era of COVID-19 [10]. This is the first case report in the published literature to share the experience of successful administration of anti-BCMA CAR T cell treatment to a patient with relapsed and refractory MM who recently recovered from COVID-19. Importantly, the administration of 1 day of LDC immediately prior to SARS-CoV-2 infection did not result in any COVID-19-related complications.

The patient experienced Grade 2 CRS, an expected toxicity associated with CAR T therapy, which resolved within 6 days, and the patient is currently in partial response (unconfirmed), per IMWG assessment [11, 12]. Inflammatory cytokines were elevated during the CRS event; however, levels were in the same range as for CAR T cell-treated patients without COVID-19 who experience CRS [13]. Hence, recent SARS-CoV-2 infection in this patient did not exacerbate CAR T-associated CRS, even though this is a toxicity known to be associated with COVID-19.

Interestingly, this patient experienced a robust humoral response to viral infection, despite his history of immune suppression. Two months after treatment with a BCMA-directed CAR T therapy, which can ablate normal BCMA expressing plasma cells, the antibody titer decreased from 1:2880 to 1:320 but remained clearly detectable approximately 4 months after infection. Whether this patient developed and retained long-term immunity against COVID-19 remains to be determined.

With the initial surge of COVID-19, many clinical trial sites paused trials of CAR T cell therapy for MM patients. As trials re-start, investigational sites should ensure access to critical care and availability of appropriate drugs to manage CAR T cell-associated toxicities and carefully weigh the potential risks and benefits of therapy prior to proceeding with CAR T cell therapy. Our report, although limited to a single patient experience, suggests that patients who have tested antibody positive for SARS-CoV-2 can proceed with CAR T cell therapy without flare-up of COVID-19-related symptoms. High antibody titers can be generated in myeloma patients; SARS-CoV-2-specific antibodies were retained despite effective anti-BCMA CAR T cell therapy in this patient. Additional studies to determine the effect of BCMA targeting agents on the risk of SARS-CoV-2 re-infection are warranted. As COVID-19 immunity after recovery has not been well characterized, appropriate precautions such as social distancing, facial mask, and good hygiene are recommended to prevent re-infection.

The information available regarding management strategies for MM patients during the COVID-19 pandemic is insufficient to provide evidence-based recommendations; however, several consensus statements have been published that provide guidance. The recommendations included within this report—securing access to supportive care, taking appropriate precautions to avoid SARS-CoV-2 infection, and carefully weighing the potential risks and benefits of CAR T cell therapy—align well with existing consensus statements [14, 15].

## Data Availability

All data are included in this case report

## Abbreviations

ANC: Absolute neutrophil count
BCMA: B-cell maturation antigen
CAR: Chimeric antigen receptor
CRP: C reactive protein
CRS: Cytokine release syndrome
IFN: Interferon
IL: Interleukin
IMWG: International Myeloma Working Group
Hb: Hemoglobin
κLC: Kappa light chain
LDC: Lymphodepleting chemotherapy
Lym: Lymphocytes
MM: Multiple myeloma
Tmax: Temperature
TNF: Tumor necrosis factor
SARS-CoV-2: Severe acute respiratory syndrome coronavirus 2
WBC: White blood cell
